# Single‐Cell Hi‐C Technologies and Computational Data Analysis

**DOI:** 10.1002/advs.202412232

**Published:** 2025-01-30

**Authors:** Madison A Dautle, Yong Chen

**Affiliations:** ^1^ Department of Biological and Biomedical Sciences Rowan University Glassboro NJ 08028 USA

**Keywords:** cell clustering, chromatin interaction, data sparsity, scHi‐C

## Abstract

Single‐cell chromatin conformation capture (scHi‐C) techniques have evolved to provide significant insights into the structural organization and regulatory mechanisms in individual cells. Although many scHi‐C protocols have been developed, they often involve intricate procedures and the resulting data are sparse, leading to computational challenges for systematic data analysis and limited applicability. This review provides a comprehensive overview, quantitative evaluation of thirteen protocols and practical guidance on computational topics. It is first assessed the efficiency of these protocols based on the total number of contacts recovered per cell and the *cis/trans* ratio. It is then provided systematic considerations for scHi‐C quality control and data imputation. Additionally, the capabilities and implementations of various analysis methods, covering cell clustering, A/B compartment calling, topologically associating domain (TAD) calling, loop calling, 3D reconstruction, scHi‐C data simulation and differential interaction analysis is summarized. It is further highlighted key computational challenges associated with the specific complexities of scHi‐C data and propose potential solutions.

## Graphical abstract

This review provides a detailed examination of scHi‐C technologies, offering quantitative evaluations of 13 protocols. It addresses computational challenges arising from data sparsity and presents systematic insights into data analysis methods, including clustering, TAD calling and 3D reconstruction. The paper also proposes practical solutions to enhance the analysis and interpretation of single‐cell chromatin architecture.

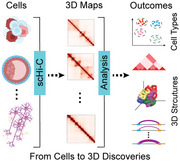

## 1 Introduction

Chromatin conformation plays a critical role in transcriptional regulation and genome replication within cells. The spatial folding of chromatin brings functionally related, yet genomically distant regions, such as enhancers, silencers and promoters, into proximity.^[^ ^1^, ^2^ ^]^ The development of DNA fluorescence in situ hybridization in the 1980s observed chromosome territories and revived the study of nuclear architecture. In 2009, Hi‐C was introduced to capture chromatin conformation on a genome‐wide scale, overcoming the resolution limitations of traditional microscopy‐based experiments.^[^ ^3^ ^]^ This method unveiled key functional structures within the genome, including A/B compartments, TADs and chromatin loops.^[^ ^3^, ^4^, ^5^ ^]^ To understand cell‐specific gene regulation and chromosomal architecture, scHi‐C was formally introduced in 2013, allowing chromatin conformation to be examined at a single‐cell level.^[^ ^6^ ^]^ Since its inception, scHi‐C technology has evolved through several key innovations, including the introduction of sorting cells into multi‐well plates, tagmentation and polymerase chain reaction (PCR). Advanced approaches, such as Dip‐C^[^ ^7^ ^]^ and sci‐Hi‐C,^[^ ^8^ ^]^ employ multiplexed tagmentation‐based strategies and combinatorial barcoding to enhance scaling and experimental efficiency. Additionally, recent developments in scHi‐C have facilitated the simultaneous generation of multi‐omics data from single‐cell experiments, encompassing methylation data, scRNA‐seq and scDNA‐seq.^[^ ^9^, ^10^, ^11^, ^12^, ^13^ ^]^

**Figure 1:**
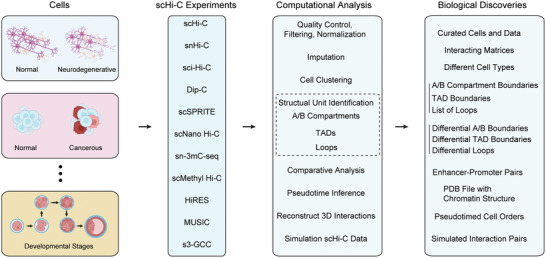
Workflow from scHi‐C experiments and computational analysis to biological discovery.

scHi‐C technologies are indispensable for a wide range of applications, such as investigating tissue heterogeneity and elucidating cellular dynamics of development pathways.^[^ ^14^, ^15^ ^]^ A typical workflow from scHi‐C experiments to biological discoveries is summarized in **Figure** **1**. scHi‐C is primarily useful for investigating cell‐specific chromosomal interactions and regulation mechanisms. For example, recent research has revealed that cohesin‐mediated loop extrusion contributes to transcription by forming a transcription elongation loop regulated by topoisomerases.^[^ ^16^ ^]^ scHi‐C is valuable for analyzing tissues with highly heterogeneous cell populations.^[^ ^13^ ^]^ For example, in brain tissue, bulk analyses often fail to capture the distinct functional profiles of various neuronal subtypes, which can be uniquely distinguished using scHi‐C coupled with scRNA‐seq.^[^ ^17^, ^18^ ^]^ A study by Liu et al. utilized scHi‐C on the adult mouse brain to map a single‐cell DNA methylome and build a 3D multi‐omics atlas, revealing subtype‐specific regulatory elements that were previously undetectable in bulk analyses.^[^ ^19^ ^]^ scHi‐C is also critical for studying rare cell types that are otherwise difficult to analyze due to limited experimental materials or overshadowing by more abundant cell populations. For instance, a snHi‐C study of mouse oocytes and zygotes, which are limited in cell numbers, revealed that chromatin architecture is uniquely reorganized during the mouse oocyte‐to‐zygote transition and is distinct in paternal and maternal nuclei within single‐cell zygotes.^[^ ^20^ ^]^ Moreover, scHi‐C offers unique insights into the dynamics of chromosomal 3D structures during cellular differentiation by providing temporal snapshots of gene transcriptional regulation among regulatory units, such as enhancers and promoters. The application of scHi‐C to track the 3D genome reorganization during early embryonic development^[^ ^21^ ^]^ highlighted the formation and dissolution of specific chromatin loops associated with key developmental genes, providing a dynamic view of how 3D genome architecture drives gene expression changes across developmental stages.

Although scHi‐C experiments provide valuable biological insights, two major challenges persist in experimental performance and downstream data analysis. First, more than ten scHi‐C protocols are currently available, each utilizing different experimental steps, leading to variable capture efficiencies. It is necessary to assess these protocols for practical selection by considering experimental complexities and data quality. Second, computational analysis of scHi‐C data faces challenges due to extreme data sparsity and selection of appropriate computational tools for various analytical tasks, such as cell clustering, A/B compartment calling, TAD calling, loop calling, 3D reconstruction, scHi‐C data simulation and differential interaction analysis. To overcome the practical barriers associated with scHi‐C techniques, we present a comprehensive review of the current protocols, datasets, and analysis pipelines for scHi‐C data. We also systematically discuss potential solutions to address the computational challenges in scHi‐C analysis.

## 2 Major Protocols of scHi‐C Experiments

**Figure 2:**
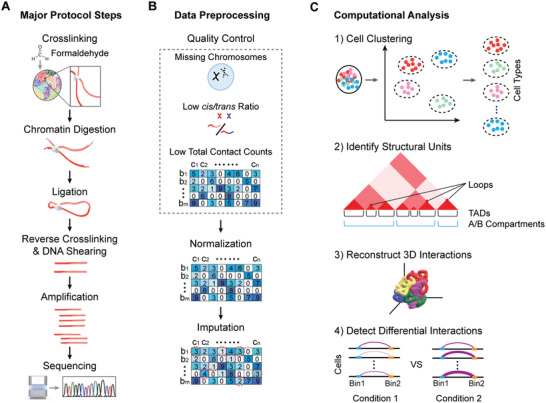
Overview of major and common steps in scHi‐C experiments A), data preprocessing B), and computational analysis topics C).

Currently, a total of thirteen scHi‐C protocols are available, either for capturing chromatin interaction signals exclusively or for combining with other assays to obtain multi‐omics data simultaneously. There are eight scHi‐C protocols available for solely obtaining chromatin interaction data (**Table** **1**). Most of these protocols involve similar major steps (**Figure** **2**A) but differ in their order and/or barcoding strategies. Nagano et al. developed the first scHi‐C protocol in 2013 and updated it in 2017.^[^ ^6^, ^15^ ^]^ Their procedure digests chromatin within intact cells, performs both biotinylated end fill and ligation before lysing the cells and isolating nuclei. Stevens et al., however, first performed cell lysis and nuclei isolation, followed by chromatin digestion, biotinylated end fill and ligation.^[^ ^22^ ^]^ Both methods are amplified using PCR but use different restriction enzymes; the Nagano et al. procedure uses *MboI*, resulting in sticky ends, while the Stevens et al. procedure uses *AluI*, resulting in blunt ends.^[^ ^15^, ^22^ ^]^ Li et al. developed another PCR‐based method, scNanoHi‐C, which removed the biotinylated end fill from the Nagano et al. procedure and instead opted for tagmentation and barcoding after reverse crosslinking, followed by pooling of cells to allow simultaneous sequencing of multiple cells.^[^ ^23^ ^]^ Zhou et al. modified Nagano et al.’s procedure by replacing the PCR amplification with whole‐genome multiple displacement amplification (MDA) and removing the biotinylated end fill steps.^[^ ^24^ ^]^ Ramani et al. developed the sci‐Hi‐C procedure to obtain scHi‐C data without the need to isolate single cells or nuclei.^[^ ^25^ ^]^ After crosslinking, nuclei are separated into a 96‐well plate where they receive their first barcode. Then, nuclei are pooled again, and chromatin is ligated before redistributing 25 nuclei per well into another 96‐well plate. A second barcode is added to each of the wells before pooling and PCR. While this method has high throughput, it makes the data analysis more complicated. Arrastia et al. developed a similar PCR‐based procedure, scSPRITE, which also does not require single cell or nuclei isolation.^[^ ^26^ ^]^ Whereas sci‐Hi‐C places a barcode between ligated ends and at the ends, scSPRITE only adds barcodes to the ends.^[^ ^8^, ^26^ ^]^ scSPRITE has three nucleus barcoding steps and three spatial barcoding steps, resulting in high costs and processing time to barcode cells.^[^ ^26^ ^]^ Tan et al. developed Dip‐C, which provides data to create haplotype‐separated Hi‐C maps.^[^ ^7^ ^]^ It removes all biotinylated end fill steps from the Nagano et al. procedure and uses multiplex end‐tagging amplification (META) instead of PCR.^[^ ^7^ ^]^ Finally, Flyamer et al. developed single nucleus Hi‐C (snHi‐C),^[^ ^20^ ^]^ the only method that isolates nuclei before chromatin fixation, but is otherwise like the protocol of Zhou et al.^[^ ^24^ ^]^

**Table 1:** Major protocols to obtain single‐cell chromatin interaction data.

Step	scHi‐C [Stevens et al. 2017]	scHi‐C [Nagano et al. 2017]	scHi‐C [Zhou et al. 2019]	snHi‐C [Flyamer et al. 2017]	sci‐Hi‐C [Ramani et al. 2017]	Dip‐C [Tan et al. 2018]	scSPRITE [Arrastia et al. 2022]	scNano Hi‐C [Li et al. 2023]
1	Cross‐linking	Cross‐linking	Cross‐linking	Nuclei isolation	Cross‐linking	Cross‐linking	Cross‐linking	Cross‐linking
2	Cell Lysis	Chromatin Digestion	Cell isolation	Crosslinking	Cell Lysis	Chromatin Digestion	Cell Lysis	Chromatin Digestion
3	Nuclei Isolation	Biotinylated End Fill	Cell Lysis	Nuclei Lysis	Chromatin Digestion	Ligation	Chromatin Digestion	Ligation
4	Chromatin Digestion	Ligation	Nuclei Lysis	Chromatin Digestion	1^st^ Round Barcoding + Addition of Bridge Adaptor	Single‐cell Isolation	Ligation	Single‐cell Isolation
5	Biotinylated End Fill	Cell Lysis	Chromatin Digestion	Ligation	Nuclei Pooling	Cell Lysis	Nuclei Isolation + In Nuclei Barcoding	Cell Lysis
6	Ligation	Nuclei Isolation	Ligation	Reverse Crosslinking	Ligation	Reverse Cross‐linking	Nuclei Pooling	Reverse Cross‐linking
7	Reverse Cross‐linking	Reverse Cross‐linking	Reverse Cross‐linking	Whole Genome Amplification (Isothermal Strand Displacement)	Dilution and Distribution	META	Nuclei Isolation + Nuclei Lysis	Tagmentation and Barcoding
8	Purification	Purification	Whole Genome Amplification (MDA)		Nuclei Lysis + Reverse Cross‐linking		Spatial Barcoding	Chromatin Pooling
9	PCR	PCR			2nd Round Barcoding		Chromatin Pooling	PCR
10					Chromatin Pooling		Reverse Crosslinking	
11					PCR		PCR	

There are five protocols that combine scHi‐C with other assays to obtain multiple omics data in parallel (**Table** **2**). For example, both single‐nucleus methyl‐3C sequencing (sn‐3mC‐seq) and scMethyl Hi‐C simultaneously gather scHi‐C data and DNA methylation state data.^[^ ^10^, ^11^ ^]^ The main difference is that scMethyl Hi‐C includes biotinylated end fill steps, while sn‐3mC‐seq does not. sn‐m3C‐seq performs a typical scHi‐C protocol with bisulfite treatment, a chemical process that converts unmethylated cytosine to uracil while leaving methylated cytosines unchanged, allowing for computational detection of methylation patterns after sequencing. The method, Hi‐C and RNA‐seq employed simultaneously (HiRES), collects both scHi‐C and scRNA‐seq data by performing reverse transcription before chromatin digestion.^[^ ^12^ ^]^ The multinucleic acid interaction mapping in single cells (MUSIC) method also jointly detects scHi‐C and scRNA‐seq data, but additionally measures RNA‐chromatin associations.^[^ ^9^ ^]^ Furthermore, the symmetrical strand sci whole‐genome plus chromatin conformation (s3‐GCC) is a PCR based method to jointly assay scHi‐C data and scDNA‐seq data.^[^ ^27^ ^]^

**Table 2:** Major protocols to obtain single‐cell multiomics data which include chromatin interaction data.

Step	sn‐3mC‐seq [Lee et al. 2019] Chromatin Interactions + Methylation State	scMethyl Hi‐C [Li et al. 2019] Chromatin Interactions + Methylation State	HiRES [Liu et al. 2023] Chromatin Interactions + RNA‐seq	MUSIC [Wen et al. 2024] Chromatin Interactions + RNA‐seq + RNA‐Chromatin associations	s3‐GCC [Mulqueen et al. 2021] Chromatin Interactions + sc‐DNA‐seq
1	Cross‐linking	Cross‐linking	Cross‐linking	Cross‐linking	Cell Lysis
2	Cell Lysis	Cell Lysis	Cell Lysis	Cell Lysis	Cross‐linking
3	Chromatin Digestion	Chromatin Digestion	Reverse Transcription	Nuclei Isolation	Chromatin Digestion
4	Ligation	Biotinylated End Fill	Chromatin Digestion	RNA Ligation	Ligation
5	Nuclei Isolation	Ligation	Ligation	Chromatin Digestion	Tagmentation
6	Bisulfite Conversion	Reverse Cross‐linking	Nuclei Isolation	DNA Ligation	Nuclei Isolation
7	Random Primed DNA Synthesis	Nuclei Isolation	Nuclei Lysis	Split‐Pool Barcoding (3 rounds)	Gap Fill + Adaptor Switching
8	Library Amplification	Bisulfite Conversion	PCR	Nuclei Lysis	PCR
9		Random Primed DNA Synthesis		Reverse Transcription	
10		Purification		Reverse Cross‐linking	
11		Library Amplification		Purification	
12				PCR	

It is worth highlighting that combining scHi‐C with other assays, such as RNA‐seq, DNA methylation or DNA sequencing, offers several significant benefits and advantages.^[^ ^9^, ^10^, ^11^ ^]^ First, combining scHi‐C with other assays provides a more comprehensive understanding of chromatin architecture and its functional outcomes. For example, pairing scHi‐C with scRNA‐seq allows researchers to link chromatin interactions directly with gene expression, offering insights into how chromatin structure influences gene regulation in individual cells.^[^ ^17^, ^18^ ^]^ Second, this combination enables the study of correlations between chromatin conformation and other molecular layers, such as epigenetic modifications within the same cell, providing insights into driving factors behind structural changes. For instance, changes in DNA methylation patterns might explain alterations in chromatin interactions observed in scHi‐C data, leading to a better understanding of epigenetic regulation.^[^ ^19^, ^28^ ^]^ Third, by combining different single‐cell assays, researchers can better dissect the complexity of heterogeneous tissues. These combined approaches are particularly valuable for detecting and studying rare cell types, as additional signals can enhance clustering accuracy.

## 3 Experimental Challenges and Potential Solutions

There are still many experimental challenges in scHi‐C protocols that limit broad application. scHi‐C protocols are highly intricate, involving multiple major steps (e.g., barcoding, digestion and ligation) that increase the risk of variability and technical noise. Many protocols, such as Nagano et al.^[^ ^6^, ^15^ ^]^ and Zhou et al.,^[^ ^24^ ^]^ are constrained by low throughput due to single‐cell isolation and individual processing steps. Methods like scSPRITE involve multiple barcoding steps, significantly increasing processing time and experimental costs.^[^ ^26^ ^]^ Differences in restriction enzymes (e.g., *MboI* versus *AluI*), barcoding strategies, and amplification techniques introduce variability, complicating data comparison across protocols. Additionally, PCR‐based methods may introduce uneven genome coverage and underrepresentation of certain genomic regions. As a result, the application of scHi‐C in biological research remains limited.

To address these challenges, several potential solutions can be implemented to improve the scHi‐C protocols and increase application. First, standardization of workflow steps, such as digestion and ligation, across protocols would reduce complexity and variability. Second, developing automated systems to streamline experimental procedures would minimize manual errors, thereby improving reproducibility. Third, adopting high‐throughput barcoding methods, such as combinatorial indexing, will increase simultaneous cell processing and reduce costs. Fourth, minimizing steps requiring expensive reagents, such as MDA, will further lower experimental costs. Finally, exploring alternative amplification methods, such as META, will ensure even genome coverage and representation, addressing one of the most significant biases in scHi‐C data. By adopting and optimizing these strategies, the scalability, reliability and utility of scHi‐C protocols are expected to improve.

## 4 Data Quality of scHi‐C Experiments

To assess the data quality of scHi‐C experiments across different protocols, we downloaded existing datasets and calculated the total number of contacts per cell and the ratio of intra‐chromosomal to inter‐chromosomal contacts (*cis/trans* ratio). The scHi‐C datasets were categorized by species and the reference genomes to which they were aligned in the original data analysis (**Table** **3**). In general, a greater number of total captured chromatin contacts is indicative of a high‐quality procedure. Additionally, more contacts between regions of the same chromosome are expected in ideal experiments, so a high *cis/trans* ratio is also indicative of good data quality.^[^ ^29^, ^30^ ^]^

**Table 3:** Statistics on chromatin interactions from existing scHi‐C datasets.

Dataset	Procedure	GSE ID	Reference Genome	Number of Cells	Interactions Mean	*cis/trans* Ratio Mean
					Median	Median
Flyamer et al. (2017)	snHi‐C	GSE80006	mm9	208	246717	9.425
					136053	8.540
			hg19	34	54316	1.998
					1417	0.428
Gassler et al. (2017)	snHi‐C	GSE100569	mm9	144	187083	12.878
					178020	12.278
Ulianov et al. (2021)	snHi‐C	GSE131811	dm3	20	66440	8.108
					52534	8.192
Stevens et al. (2017)	scHi‐C (Stevens 2017)	GSE80280	mm10	8	60967	12.136
					61095	13.268
Nagano et al. (2013)	scHi‐C (Nagano 2013)	GSE48262	mm9	10	232452	10.462
					225765	10.608
Nagano et al. (2017)	scHi‐C (Nagano 2017)	GSE94489	mm9	3882	225687	7.168
					179224	6.545
Collombet et al. (2020)	scHi‐C (Nagano 2017)	GSE129029	mm10	1015	268836	14.565
					224129	10.577
Rappoport et al. (2023)	scHi‐C (Nagano 2017)	GSE148793	mm9	3648	112422	17.696
					101273	15.264
Zhou et al. (2019)	scHi‐C (Zhou 2019)	GSE123109	rgap7	15	7407	7.651
					5184	5.592
Ramani et al. (2017)	sci‐Hi‐C	GSE84920	mm10	2624	5534	4.386
					3397	4.189
			hg19	2972	9054	6.430
					4900	5.741
			Mixed (mm10, hg19)	6333	19248	5.909
					11440	5.153
Kim et al. (2020)	sci‐Hi‐C	⧫	hg19	19388	5509	5.192
					3683	4.096
Tan et al. (2018)	Dip‐C	GSE117876	hg19	35	937763	2.552
					874453	2.415
Tan et al. (2019)	Dip‐C	GSE121791	mm10	409	252292	2.594
					252348	0.992
Tan et al. (2021)	Dip‐C	GSE162511	mm10	3646	365076	5.416
					363691	4.676
Arrastia et al. (2022)	scSPRITE	GSE154353	Mixed (mm9, hg19)	1000	176982	5.88
					78870	5.49
Li et al. (2023)	scNanoHi‐C	GSE217189	mm10	672	800199	10.129
					800278	10.211
			hg38	1077	644515	7.029
					646030	6.199
			Mixed (mm10, hg38)	96	405589	6.143
					380795	6.163
Lee et al. (2019)	sn‐m3C‐seq	GSE124391	mm10	540	845132	6.017
					710549	5.993
			hg38	172	620957	5.775
					555807	5.944
Luo et al. (2019)	sn‐m3C‐seq	GSE130711	hg19	4238	1247004	8.843
					1271124	6.874
Li et al. (2019)	scMethyl Hi‐C	GSE119171	mm9	150	77811	3.698
					73548	2.813
Mulqueen et al. (2021)	s3‐GCC	GSE174226	hg38	211	1199765	15.006
					808076	14.297
Liu et al. (2023)	HiRES	GSE223917	mm10	7868	279855	6.558
					270394	5.223
Wen et al. (2024)	MUSIC	GSE253754	mm10	1238	84228	1.991
					43369	2.004
			hg38	10366 (AD) +2267 (H1)	92746	0.912
					30525	0.195

⧫No GEO Accession ID; access at https://noble.gs.washington.edu/proj/schic‐topic‐model/

**Figure 3:**
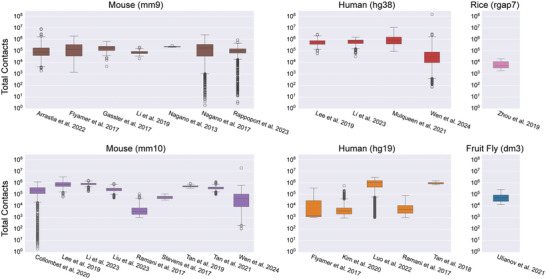
Total number of detected chromatin interactions for different datasets. Information details about each dataset can be found in Table 3.

**Figure 4:**
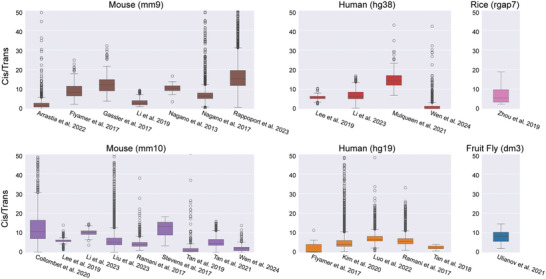
Ratio of detected *cis* chromatin interactions to detected *trans* chromatin interactions. Information details about each dataset are listed in Table 3.

**Figure 5:**
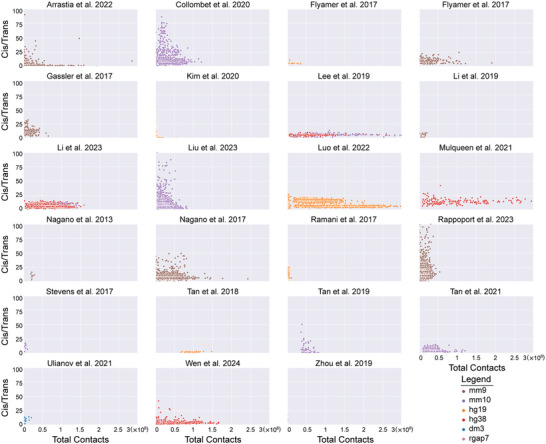
*Cis/trans* ratio as a function of number of total contacts per cell by dataset. Information details about each dataset are listed in Table 3. Each dot represents one cell. Cells are colored according to cell type based on the reference genome used for mapping in the original analysis.

We first evaluated the quality of the datasets generated from scHi‐C protocols designed to solely detect chromatin interactions but not integrate other data types. The datasets from Ramani et al. 2017^[^ ^8^ ^]^ and Kim et al. 2020,^[^ ^31^ ^]^ both utilizing the sci‐Hi‐C protocol, exhibit high *cis/trans* averages and medians but have low numbers of total contacts (**Figure** **3** and **Figure** **4**). The scSPRITE protocol, which generated the dataset from Arrastia et al. 2022,^[^ ^26^ ^]^ performs similarly to the sci‐Hi‐C protocol by these metrics. The snHi‐C protocol, used for the datasets from Flyamer et al. 2017,^[^ ^20^ ^]^ Gassler et al. 2017,^[^ ^32^ ^]^ and Ulianov et al. 2021,^[^ ^33^ ^]^ performs about average for experiments using mouse and fruit fly cells, but it does not perform well on human cells, with a median *cis/trans* ratio of 0.428. The Stevens et al. 2017 protocol,^[^ ^22^ ^]^ has a high mean *cis/trans* ratio of 12.136 and a median *cis/trans* ratio of 13.268, but it recovers only about a quarter of the total contacts compared to the Nagano et al. 2013 protocol^[^ ^6^ ^]^ or the Nagano et al. 2017 protocol.^[^ ^15^ ^]^ The datasets from Tan et al. 2018,^[^ ^7^ ^]^ Tan et al. 2019^[^ ^34^ ^]^ and Tan et al. 2021,^[^ ^35^ ^]^ generated using Dip‐C, show an average recovery of contacts, but have some of the lowest *cis/trans* ratios among all the datasets. scNanoHi‐C^[^ ^23^ ^]^ is another protocol that is demonstrated to work on both mouse and human cells. It recovers a higher‐than‐average number of total contacts and has a *cis/trans* ratio comparable to other methods (Table 3, **Figure** **5**). In summary, we observed that Nagano et al. 2017 protocol and scNanoHi‐C have higher total captured contacts and *cis/trans* ratios among those scHi‐C protocols focusing on solely interaction detection.

We then assessed the quality of datasets generated by the protocols that combine scHi‐C with other assays. The sn‐m3c‐seq protocol was employed to generate two datasets: one by Lee et al. 2019^[^ ^10^ ^]^ and another by Luo et al. 2022.^[^ ^17^ ^]^ Although the same protocol was employed, the early application on 540 mouse cells and 172 human cells in Lee's research generated lower mean and median numbers of total contacts per cell, nearly half of what was observed in the late application on 4238 human cells in Luo's research (Table 3). This difference may be attributed to experimental performance or deep sequencing depth. Another protocol that measures chromatin interactions and methylation state simultaneously, scMethyl Hi‐C, was used to generate the Li et al. 2019 dataset.^[^ ^11^ ^]^ However, this dataset recovers only about a tenth of the contacts recovered using the sn‐m3C‐seq protocol. The HiRES protocol^[^ ^12^ ^]^ performs similarly to the Nagano et al. 2017 protocol^[^ ^15^ ^]^ while also collecting RNA‐seq data. The s3‐GCC protocol^[^ ^27^ ^]^ demonstrates the best consistency in the *cis/trans* ratios, maintaining stable ratios even when the total number of recovered contacts varies. This protocol also captures an above‐average total number of contacts, though it has only been used once by Mulqueen et al.^[^ ^27^ ^]^

We compared the performance of datasets generated by scHi‐C alone and those generated by multi‐omics scHi‐C protocols. However, no significant differences were found in either the total number of captured contacts or the *cis/trans* ratios (Wilcoxon‐Mann‐Whitney U‐test, P value cutoff of 0.05). This result suggests that multi‐omics scHi‐C experiments, such as sn‐m3C‐seq, can efficiently capture chromatin interactions while also detecting other molecular signals within the same cell. In conclusion, we recommend multi‐omics scHi‐C protocols as effective experiments for simultaneously detecting chromatin interactions and other molecular signals.

## 5 Quality Control and Pre‐Processing of scHi‐C Data

### 5.1 Quality Control and Filtration

As scHi‐C involves intricate procedures, each step potentially introduces errors or inefficiencies that lead to incomplete or low‐quality data, careful quality control and filtration are critical for reliable downstream analyses (Figure 2B). One common quality control method requires users to remove cells with a contact count below a specified threshold; there is no literature value, different studies use values from 1000 to 5000 contacts per cell^[^ ^31^, ^36^, ^37^, ^38^ ^]^ (**Table** **4**). Other methods include removing cells with a *cis/trans* chromosomal contact ratio that falls below 1, cells with a uniquely mapped read percentage lower than 95%, cells with a low proportion of short‐range contacts to long‐range contacts, and cells missing interactions from any chromosome. However, these filtration methods are static and empirical; they do not assess quality control in relation to the contact coverage.

**Table 4:** Preprocessing methods for scHi‐C data.

Algorithm	Data Imputation	Filtering	Normalization	PMID	Publication Date
scHiCluster	Random walk with restart	Cells have a minimum of 5k contacts, each chromosome has more contacts than the length in Mb, and all chromosomes are present in the file (‡)	✦	31235599	05.2019
scHiC Topics	Latent Dirichlet allocation	Minimum of 1000 unique reads, *cis/trans*‐chromosomal contact ratio greater than 1, and uniquely mapped read percentage greater than 95% (‡)	✦	32946435	09.2020
scVI‐3D	Deep generative model and variational autoencoders	Recommended filtering by dataset source, recommended 1Mb resolution (‡)	✦	36253828	03.2021
scHiCTools	1. Linear Convolution 2. Random walk 3. Network enhancing	Threshold for cells with a high proportion of short‐range contacts (<2Mb) to mid‐range contacts (2‐12Mb)	✦	34003823	05.2021
Higashi	Hypergraph representation learning	Minimum 2000 read pairs for 500Kb resolution (‡)	✦	34635838	02.2022
scHiCEmbed	Random walk with restart	Minimum 5000 read pairs for 500Kb resolution (‡)	✦	35741810	06.2022
HiCImpute	Bayesian hierarchical model	Recommended filtering by dataset source (‡)	✦	35696429	06.2022
SnapHiC2	Sliding window (approximates random walk with restart)	Recommended filtering by dataset source (‡)	✦	35685374	06.2022
Fast‐Higashi	Partial random walk with restart and tensor decomposition model	Recommended filtering by dataset source (‡)	✦	36265466	10.2022
HiCS	Biased random walk (Node2Vec)	Minimum of 250000 contacts (‡)	✦	36658736	01.2023
scHiCEDRN	Deep Learning (GAN Framework)	Recommended filtering by dataset source (‡)	✦	37498561	07.2023
scHiCDiff	Gaussian convolution	Minimum of 5000 contacts (‡)	✦ scHiCNorm	37847655	10.2023

✦Integrated into Data Imputation, ‡ Performed before utilizing algorithms

Currently, GiniQC is the only package available to perform quality control and filtration that accounts for the types of contacts present and their relative coverage.^[^ ^39^ ^]^ GiniQC can assess the spread of *trans* chromosomal contacts. Since chromosomes only contact a limited number of other chromosomes in specific locations, *trans* contacts should vary highly between bins and be sparse. Therefore, GiniQC measures the clumping of contacts to determine a filtration threshold. Galaxy HiCExplorer^[^ ^40^ ^]^ filters cells using a three‐step process: 1) only matrices which contain all listed chromosomes are kept, 2) only matrices which have a minimum read coverage are accepted where the minimum read coverage is set by the user, and 3) the matrix must have a minimum density of recorded data points close to the main diagonal. Although Galaxy HiCExplorer offers a straightforward, user‐friendly filtration process, it may lack the ability to account for nuanced data characteristics like the spread of trans chromosomal contacts.

### 5.2 Data Normalization

Many of the packages that perform data normalization combine it with their data imputation step (Table 4). scHiCNorm normalizes *cis* contacts by calculating local bias and using it as a feature in multiple distribution fitting models.^[^ ^41^ ^]^ The model with the best fit is then used to normalize the scHi‐C data. Precalculated local bias values are provided for the mm9, mm10, hg19 and hg38 reference genomes. Perl scripts are available to calculate local bias values for other reference genomes. Following the calculation of local bias, normalization is applied for *cis* contacts; however, this pipeline does not normalize *trans* contacts due to their sparsity. BandNorm uses band transformation to normalize data, following a multi‐step procedure that removes genomic distance bias, normalizes contacts based on sequencing depth, and applies a band‐dependent contact decay estimate.^[^ ^42^ ^]^ BandNorm is implemented in R and offers a computationally efficient normalization of scHi‐C data. Galaxy HiCExplorer 3 offers a two‐step normalization procedure. Users can choose one of the following initial normalization options: 1) normalization to the read coverage of all given matrices, 2) row sum normalization, or 3) multiplication by a customized value. This is followed by Knight‐Ruiz normalization to produce the final normalized matrices.^[^ ^40^ ^]^ While this is useful for datasets with many matrices, these normalization procedures are standard and do not incorporate local bias information as chicory and BandNorm do.^[^ ^40^, ^41^, ^42^ ^]^

### 5.3 Data Imputation

Due to the experimental complexity of scHi‐C experiments, the data often have a significant level of dropouts and noise. Here, data imputation mainly refers to the method used to infer missing interactions in the scHi‐C data. The most popular approach for data imputation in scHi‐C data is the random walk. scHiCluster^[^ ^43^ ^]^ and scHiCEmbed^[^ ^36^ ^]^ employ a random walk with restart, Fast‐Higashi uses a partial random walk combined with a tensor decomposition model,^[^ ^37^, ^44^ ^]^ SnapHiC2 approximates a random walk by using a sliding window,^[^ ^45^ ^]^ HiCS imputes data using a biased random walk,^[^ ^46^ ^]^ and scHiCTools allows users to choose between linear convolution and two forms of random walk implementation.^[^ ^47^ ^]^ Specifically, the random walk leverages information from neighboring cells to move from a starting node to another node following certain probabilities. The stable distribution function between two points can be found and used to impute the missing data.^[^ ^48^ ^]^ Random walk is easy to implement and does not assume any specific data distribution. However, it is biased toward observations from surrounding data and could be heavily impacted by missing data. As scHi‐C data collection is not yet optimized to collect all available contacts, random walks may result in a high degree of noise and improperly imputed data.

Gaussian convolution is used by scHiCDiff to smooth the scHi‐C data.^[^ ^49^ ^]^ This approach assumes that cellular locations in closer proximity have a greater weight than more distant regions, which aligns with expectations in chromatin interactions. However, it may improperly smooth regions with high frequency contacts, such as heterochromatin regions. HiCImpute employs a Bayesian hierarchical model for data imputation.^[^ ^50^ ^]^ Bayesian inference is advantageous because it naturally integrates new information with prior knowledge, allowing the model to adapt to additional data. Although prior knowledge is necessary to initialize the analysis, its influence diminishes as more data is incorporated, making this method well‐suited for the large size of scHi‐C datasets.

Deep learning‐based strategies are employed by scVI‐3D,^[^ ^42^ ^]^ scHiCEDRN^[^ ^51^ ^]^ and Higashi^[^ ^37^ ^]^ (Table 4). These approaches eliminate the need for the prior knowledge required by Bayesian hierarchal inference; however, they require significant computational power, making them more challenging to implement and slower than other methods. scHiC Topics utilizes latent Dirichlet allocation (LDA), a form of unsupervised machine learning, where groups of chromatin contacts are treated as words.^[^ ^31^ ^]^ This approach allows for imputation without prior information but requires careful preprocessing as LDA is highly sensitive to data preprocessing.

## 6 scHi‐C Data Analysis Topics

Divergent computational analyses are required to fully understand the biological insights of chromatin structure through use of scHi‐C data. The primary topics include cell clustering, A/B compartment calling, TAD calling, loop calling, 3D interaction reconstruction, comparative analysis of chromatin interactions and scHi‐C data simulation (Figure 1 and Figure 2C). Currently, several tools are available for these topics, but most only address one topic, with few addressing multiple topics simultaneously (**Table** **5**).

**Table 5:** Pipeline capabilities to analyze scHi‐C data.

Pipeline	Cell Clustering	A/B Compartment Calling	TAD Calling	Loop Calling	Reconstruct 3D Interactions	Detect Differential Interactions	Simulate scHi‐C Data	Pseudotime Inference	PMID	Publication Date
Higashi	✓	✓	✓	✗	✗	✗	✗	✗	34635838	02.2022
scHiCEmbed	✓	✗	✓	✗	✓	✗	✗	✗	35741810	06.2022
scHiCluster	✓	✗	✗	✗	✗	✗	✗	✗	31235599	05.2019
Galaxy HiCExplorer 3	✓	✗	✗	✗	✗	✗	✗	✗	29901812	03.2020
scHiCTools	✓	✗	✗	✗	✗	✗	✗	✗	34003823	05.2021
BandNorm and scGAD	✓	✗	✗	✗	✗	✗	✗	✗	36253828	03.2021
scVI‐3D	✓	✗	✗	✗	✗	✗	✗	✗	36253828	03.2021
HiCImpute	✓	✗	✗	✗	✗	✗	✗	✗	35696429	06.2022
Fast‐Higashi	✓	✗	✗	✗	✗	✗	✗	✗	36265466	10.2022
scHiCEDRN	✓	✗	✗	✗	✗	✗	✗	✗	37498561	07.2023
scGHOST	✗	✓	✗	✗	✗	✗	✗	✗	38589516	08.2024
HiCS	✗	✗	✓	✗	✗	✗	✗	✗	36658736	01.2023
DeDoc2	✗	✗	✓	✗	✗	✗	✗	✗	37162225	05.2023
SnapHiC2	✗	✗	✗	✓	✗	✗	✗	✗	35685374	06.2022
NucProcess + NucDynamics	✗	✗	✗	✗	✓	✗	✗	✗	28289288	04.2017
Si‐C	✗	✗	✗	✗	✓	✗	✗	✗	34272403	07.2021
DPDchrom	✗	✗	✗	✗	✓	✗	✗	✗	34793453	11.2021
scHiCDiff	✗	✗	✗	✗	✗	✓	✗	✗	37847655	10.2023
scHi‐CSim	✗	✗	✗	✗	✗	✗	✓	✗	36708167	06.2023
scHiCPTR	✗	✗	✗	✗	✗	✗	✗	✓	36205615	10.2022

### 6.1 Cell Clustering

Cell clustering is a strategy to group similar cells, serving as a critical step for downstream cell type‐specific analyses. There are ten existing analysis pipelines for clustering scHi‐C data. Higashi allows users to perform cell clustering, A/B compartment calling, and TAD calling all within the same framework, but requires a significant amount of input information^[^ ^37^ ^]^ (**Table** **6**). They also provide a Fast‐Higashi implementation, but Fast‐Higashi only performs cell clustering. Users are required to feed the output of Fast‐Higashi to Higashi for A/B compartment calling and TAD calling.^[^ ^37^, ^44^ ^]^ scHiCEmbed also allows users to perform multiple analyses in the same framework, specifically cell clustering, TAD calling and 3D reconstruction.^[^ ^36^ ^]^ It requires binning of the input data, so careful selection of the bin size should be considered.^[^ ^36^, ^37^ ^]^ scHiCluster allows users to process multiple cells without writing their own function call by taking a tab‐separated file containing the cell names and file paths to contact matrices and chromosome sizes as input.^[^ ^43^ ^]^ Galaxy HiCExplorer 3 is unique because it provides a graphical user interface when running the commands in Galaxy, thus allowing users to process their data without extensive programming knowledge^[^ ^40^ ^]^ (Table 6). scHiCTools allows users to cluster cells using either contact pairs or matrices and an annotated reference genome.^[^ ^47^ ^]^ BandNorm and scGAD use a weighted contact pairs file, and gene locations file to provide both cell clustering and gene associating domains, providing the only conversion between scHi‐C data and gene expression data.^[^ ^42^ ^]^ The researchers also provide a separate package, scVI‐3D, which takes weighted contact pairs files and a chromosome sizes file to normalize and cluster cells.^[^ ^42^ ^]^ HiCImpute requires the least amount of information, only requiring contact matrices of the single cells to provide cell clustering annotations.^[^ ^50^ ^]^ Finally, scHiCEDRN provides a deep learning‐based cell clustering analysis; however, the pipeline is not optimized to process user input.^[^ ^51^ ^]^

**Table 6:** Cell clustering methods for scHi‐C data analysis.

Algorithm	Data Input Structure	Data Output Structure	Language
BandNorm and scGAD	Contact pairs file (.tsv) including locations and weight or. hic file, and gene starting and ending coordinates file	Cell cluster annotations, normalized contact files, and scGAD scores	R
Fast‐Higashi	JSON file with selected parameters, reference genome, reference genome cytoband file, scHi‐C data in Higashi_v1 or Higashi_v2 format, and Python pickle file for cell labels	Dictionary of cell cluster annotations	Python
Galaxy HiCExplorer 3	.scool file	Cell cluster annotations, interaction matrix, and 2D contact map	Python or Galaxy
HiCImpute	A list of 2D, single‐cell contact matrices	Cell cluster annotations	R
Higashi	JSON file with selected parameters, reference genome, reference genome cytoband file, scHi‐C data in Higashi_v1 or Higashi_v2 format, and Python pickle file for cell labels	Imputed contact matrix, cell cluster annotations, TAD calling and insulation (.hdf5), and A/B compartments (.hdf5)	Python
scHiCEDRN	Only prepared to use provided datasets	.mcool files	Python
scHiCEmbed	One single‐cell Hi‐C matrix (format: bin_index1 bin_index2 contact_counts) and bin index file (with columns chromosome_id, chromosome_length, bin_index_start, and bin_index_end)	Embedding matrix and TAD boundary file (.bed) (PDB file *Perl required)	Python or R
scHiCluster	Table with cell names and file paths (.tsv) to individual cell contact matrix files (.hic), and chromosome sizes	Cell cluster annotations	Python
scHiCTools	Contact pairs files or cell contact matrices and reference genome	Imputed contact matrix (numpy array) and cell clustering annotations (numpy array)	Python
scVI‐3D	Chromosome sizes (.tsv) and contact pairs file (.tsv) including locations and weight	Cell cluster annotations, normalized scHi‐C data (.tsv), and t‐SNE and UMAP projection figures	Python

Although cell clustering methods are available for scHi‐C data, there is still considerable room for improvement. scHi‐C data presents unique challenges for cell clustering due to inherent variability, high dimensionality and data sparsity.^[^ ^52^ ^]^ While clustering based directly on contacts from single cells is feasible and does not require a prior analysis step, clustering results may be enhanced if clusters are identified at different scales, such as A/B compartments or TADs. Additionally, applying advanced deep‐learning strategies could offer both dimensionality reduction and better consideration of inherent variability.^[^ ^53^ ^]^

### 6.2 A/B Compartment Calling

A/B compartments segregate active chromatin regions, known as A compartments, from inactive regions, known as B compartments.^[^ ^3^, ^54^ ^]^ A/B compartments are dynamic structures which undergo significant changes during cell differentiation, making A/B compartments distinct to each cell type.^[^ ^53^, ^54^, ^55^, ^56^ ^]^ The frequency of chromatin interactions captured by Hi‐C or scHi‐C data is a primary signal to predict A/B compartments. Regions with higher interaction frequencies within themselves are more likely to belong to the same compartment, while the interaction patterns between A and B compartments are usually weaker. Furthermore, the rate at which interaction frequencies decrease with increasing genomic distance (contact decay) can differ between A and B compartments. A compartments often have a slower decay rate, indicating more frequent long‐range interactions within active regions, while B compartments have a faster decay rate, reflecting more localized interactions within inactive regions.

These statistical features can be used for separating A and B compartments and many computational tools are available to perform this analysis (Table 5). For the Higashi analysis suite, A/B compartment calling is included, making it one of the few packages that complete multiple computational tasks within a single framework. Higashi requires a reference genome, a reference genome cytoband file, scHi‐C data in the Higashi formats, a Python pickle file containing cell labels, and a JSON file with file paths and selected parameters. From this, Higashi produces an imputed contact matrix, cell cluster annotations, TAD calling and insulation positions in an. hdf5 file format, and the A/B compartment boundaries and classifications in an. hdf5 file format.^[^ ^37^ ^]^ The Higashi imputation results, embedding results, and the A/B compartment insulation scores, are used as input for scGHOST.^[^ ^57^ ^]^ scGHOST utilizes a graph‐embedded neural network with a constrained random walk to identify sub‐compartments from intra‐chromosomal contacts, and it has been shown to reveal cell type‐ and allele‐specific sub‐compartment links. scGHOST further demarcates these compartments into their sub‐compartments, providing a. bed file with the compartments and a small script to visualize the compartments on a UMAP. While scGHOST can be run independently of Higashi, it is designed for use after Higashi.

### 6.3 TAD Calling

TADs help to organize chromatin within A/B compartments by creating additional boundaries that enable precise transcriptional regulation.^[^ ^4^, ^58^ ^]^ Similar to A/B compartments, interactions are more likely to occur within the same TAD than across TAD boundaries.^[^ ^59^, ^60^, ^61^ ^]^ There are currently four methods to infer TADs from scHi‐C data. Two of these, Higashi and scHiCEmbed, offer the capability to perform multiple analyses within a single framework. Higashi allows users to perform cell clustering, A/B compartment calling and TAD calling, while scHiCEmbed supports cell clustering, TAD calling and 3D chromatin reconstruction.^[^ ^36^, ^37^ ^]^ scHiCEmbed requires less data input compared to Higashi, needing only a scHi‐C matrix and a bin index file (**Table** **7**). HiCS is another tool that performs TAD calling and allows for processing of multiple cells simultaneously by placing all chromatin contacts files and a chromosome size file into one folder. However, it does not accept condensed data formats, such as. hic or. cool files, and requires each chromatin contact to be represented on its own line, in the style of the. pairs format.^[^ ^46^ ^]^ Finally, DeDoc2 calls TAD boundaries using only a contact matrix, and requires use of Java rather than Python or R.^[^ ^62^ ^]^

**Table 7:** TAD calling methods for scHi‐C data analysis.

Algorithm	Data Input Structure	Data Output Structure	Language
Higashi	JSON file with selected parameters, reference genome, reference genome cytoband file, scHi‐C data in Higashi_v1 or Higashi_v2 format, and Python pickle file for cell labels	Imputed contact matrix, cell cluster annotations, TAD calling and insulation (.hdf5), and A/B compartments (.hdf5)	Python
scHiCEmbed	One single‐cell Hi‐C matrix (format: bin_index1 bin_index2 contact_counts) and bin index file (with columns chromosome_id, chromosome_length, bin_index_start, and bin_index_end)	Embedding matrix and TAD boundary file (.bed) (PDB file *Perl required)	Python or R
HiCS	Chromosome sizes and contact files (.tsv) – all in the same directory, one file per cell, one line per contact	Imputed contacts file, TAD boundaries file	Python
DeDoc2	Contact matrix (n x n, tab delimited) or sparse matrix (number of bins, from_bin, to_bin, edge_weight)	TAD boundaries file	Java

### 6.4 Loop Calling

Chromatin loops are highly dynamic structures that organize chromatin into smaller functional units than TADs.^[^ ^5^, ^63^ ^]^ The formation and location of these loops are typically controlled by the interaction between cohesin and CTCF, which together form a complex that establishes physical boundaries.^[^ ^64^ ^]^ Chromatin loops play crucial roles in transcriptional activation, repression and DNA recombination.^[^ ^65^ ^]^ SnapHiC2 is currently the only analysis pipeline designed to call loops from scHi‐C data^[^ ^45^ ^]^ (Table 5). It can identify loops with a resolution of up to 5 kb resolution and allow for loop calling without data imputation. SnapHiC2 requires three files as input: 1) tab‐separated files containing the mapped read pairs for each single cell, 2) a chromosome sizes file for the reference genome, and 3) a binned bed file of the genomic regions that are excluded from loop calling. The pipeline outputs a. bedpe file for the identified chromatin loops with strength scores, and a. bedpe file for the loop candidates with strength scores. Optionally, SnapHiC2 can also generate. hic and. cool files, which are useful for visualization.^[^ ^45^ ^]^

### 6.5 Reconstruct 3D Chromatin Structure

Chromatin contacts captured by scHi‐C data can be used to reconstruct the 3D structure of chromatin, providing a comprehensive view of how chromatin is spatially organized within the nucleus.^[^ ^22^, ^36^, ^66^, ^67^ ^]^ This reconstruction is essential for visualizing where key chromatin structures, such as A/B compartments, TADs and loops, are located within the 3D space of the nucleus. It is also valuable for studying the interactions between chromatin and other nuclear components, such as nuclear bodies or the nuclear lamina, which can influence chromatin dynamics and gene regulation.

There are several pipelines available for reconstructing chromatin interactions in 3D. Among them, NucProcess provides a comprehensive solution by taking paired FASTQ files and a reference genome to generate a PDB file, which is a format compatible with molecular visualization software^[^ ^22^ ^]^ (**Table** **8**). This pipeline integrates both data processing and visualization, offering a streamlined workflow. Another tool, scHiCEmbed, also generates a PDB file but requires the scHi‐C data to be processed into a specific format and involves the use of Perl.^[^ ^36^ ^]^ However, scHiCEmbed offers additional functionality by allowing users to perform cell clustering and TAD calling within the same framework, making it a versatile option for multi‐faceted analysis. Si‐C stands out as the only BASH‐based program in this context, requiring a list of chromosome lengths and chromosome contact pairs to produce a PDB file.^[^ ^66^ ^]^ Its simplicity and reliance on BASH make it accessible for users familiar with basic command‐line operations. DPDChrom can produce a Mol2 file from a weighted paired contact file, which is another format used for molecular modeling.^[^ ^67^ ^]^ However, this pipeline requires the use of Fortran in addition to Python, adding a layer of complexity to its implementation.

**Table 8:** 3D reconstruction methods for scHi‐C data analysis.

Algorithm	Data Input Structure	Data Output Structure	Language
NucProcess + NucDynamics	Paired FASTQ file and reference genome	PDB file	Python
scHiCEmbed	One single‐cell Hi‐C matrix (format: bin_index1 bin_index2 contact_counts) and bin index file (with columns chromosome_id, chromosome_length, bin_index_start, and bin_index_end)	Embedding matrix and TAD boundary file (.bed), PDB file	Python or R + Perl
Si‐C	List of chromosome lengths and chromosome contact pairs (.tsv) in the format (chr_A pos_A chr_B pos_B)	PDB file	BASH
DPDChrom	Weighted pair reads file in the format (chr1, chr2, start1, end1, start2, end2, count)	Mol2 file	Fortran + Python

### 6.6 Comparative Analysis of Chromatin Contacts

**Figure 6:**
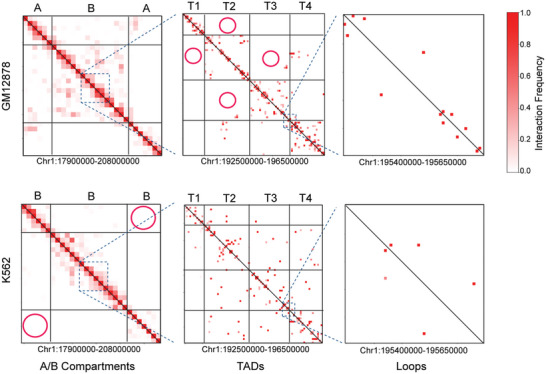
Distinct chromosomal organizations in GM12878 and K562 cells across multiple scales. The A/B compartments and TADs are annotated using the authors' original annotations.^[^ ^23^ ^]^ The red circles highlight significantly differentially interacting regions within A/B compartments and TADs between two cell lines.

Comparative analysis of chromatin contacts is crucial for identifying contact variations across different conditions.^[^ ^49^ ^]^ Such analyses can reveal distinctions among cell types, functional alterations between normal and diseased states, disease progression and developmental changes. For example, **Figure** **6** shows a chromosomal region (chr1:179000000‐208000000) for GM12878 and K562 cell lines that have distinct chromosomal organizations at multiple scales of A/B compartments, TADs and chromatin loops.^[^ ^23^ ^]^

scHiCDiff is an R‐based tool that identifies differential chromatin interactions.^[^ ^49^ ^]^ It requires a normalized matrix where the rows represent bin and chromosome numbers, and the columns represent cells and conditions. scHiCDiff utilizes negative binomial (NB) distributions to model interaction strengths among chromosomal loci and applies zero‐inflated NB (ZINB) distributions to handle sparse data among cells, where significant dropout noise results in artificially observed zeros representing undetected interactions among bins.^[^ ^49^ ^]^ scHiCDiff then compares the distributions of each interaction bin pair between two conditions using non‐parametric approaches, such as the Cramér–von Mises (CVM) test and the Kolmogorov–Smirnov (KS) test. The null hypothesis is that the two groups of single cells are from the same population, meaning that the contact counts at each bin pair are drawn from the same distribution. Consequently, hypothesis testing is conducted through the KS or CVM tests for the non‐parametric methods. scHiCDiff reports the positions of these differential interactions and P values. While this bin‐based comparison method is straightforward, it cannot detect changes at different scales, such as A/B compartments, TADs and loops.

### 6.7 Pseudotime Inference

Pseudotime inference, also known as trajectory inference, is a critical computational technique that leverages single‐cell data to uncover spatial and temporal variations in cellular dynamics. Currently, scHiCPTR is the only available analysis package for this type of analysis by using scHi‐C data.^[^ ^68^ ^]^ scHiCPTR is an unsupervised graph‐based method that infers one set of edges from a hybrid index combining the dice coefficient and betweenness centrality, while another set of edges is derived by projecting a Euclidean distance‐based K‐nearest neighbor graph onto a minimum spanning tree, simultaneously. The package is implemented in Python and requires a contact matrix with variable bin sizes for intra‐chromosomal contacts, along with the chromosome sizes of the reference genome. Users have the option to impute data using a convolutional random walk. The output provides the pseudotime inference value for each cell, and a short script is included for visualizing the results.

### 6.8 Simulate scHi‐C Data

When benchmarking both new and existing pipelines, simulated data is particularly beneficial because the true interactions are generally known, allowing for verification. Currently, scHi‐CSim is the only package available to simulate scHi‐C data (Table 5). scHi‐CSim adjusts sequencing depth and cell number by using real scHi‐C data as a seed. Since actual scHi‐C data is still needed as a reference, this pipeline is primarily useful for assessing the performance and robustness of existing pipelines against data sparsity and noise.^[^ ^38^ ^]^

## 7 Computational Challenges and Future Research

There is no doubt that these computational methods have provided significant convenience in analyzing scHi‐C data. However, due to the complexity of scHi‐C protocols and the low data quality, significant computational challenges remain in improving the performance of current analyses. Additionally, novel computational analyses are required to fully understand the biological significance of chromatin structure.

### 7.1 Experimental Design and Data Sparsity

**Figure 7:**
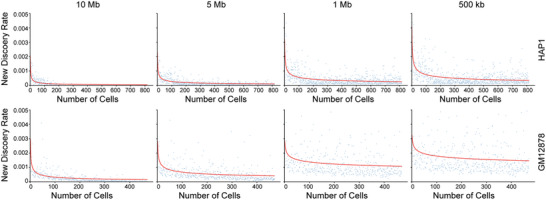
Discovery rates across varying bin sizes and cell numbers for HAP1 and GM12878 cells. Data for 812 HAP1 cells and 471 GM12878 cells from the Ramani et al. 2017 dataset were analyzed at different bin sizes.^[^ ^8^ ^]^ The red line represents the fitted curve based on power law functions.

The limitations of scHi‐C protocols and sequencing steps mean that uniquely captured paired‐end reads for each cell represent only a small fraction of the theoretically possible chromatin interactions, leading to extremely sparse interaction maps. From a sequencing budget perspective, there are constraints on either increasing sequencing depth per cell or increasing the number of cells analyzed. Therefore, when a scHi‐C experiment is completed, it is beneficial to pre‐sequence a small number of cells to 1) determine the theoretical maximum of unique chromatin interactions that can be captured per cell, 2) assess whether additional sequencing is necessary to capture more novel chromatin interactions and 3) estimate the sufficient number of cells required to adequately sample the interactions for a specific cell type. To demonstrate the impact of cell numbers on capturing novel chromatin interactions, we calculated the new discovery rate, defined as the proportion of newly detected contacts for bin pairs per cell among the total detected contacts across all cells. The results on HAP1 and GM12878 cell lines^[^ ^8^ ^]^ demonstrate a clear decay in the new discovery rates as more cells are included (**Figure** **7**). Specifically, the fitted decay curves for 812 HAP1 cells show better saturation compared to 471 GM12878 cells, indicating that more GM12878 cells may be required to achieve a similar level of saturation. Additionally, the saturation is achieved with fewer cells when larger bin sizes are used for both cell lines, highlighting the influence of cell numbers and bin sizes on chromatin interaction detection and saturation.

Unfortunately, there are no quantitative models available for precise saturation analysis for different datasets as the captured chromatin interactions are influenced by a complex interplay of experimental materials, protocol procedures, and sequencing constraints. To address this limitation, a promising strategy is to design statistical models based on stochastic process theory to analyze the saturation status of scHi‐C data.^[^ ^69^ ^]^ These statistical models should effectively mimic the key and common steps in various scHi‐C experiments and incorporate multiple parameters to evaluate the effects of sequencing depth, cell numbers and the number of theoretical chromatin interactions in each experiment. Consequently, such models will help systematically access the saturation status of currently available scHi‐C dataset, providing valuable feedback to biologists regarding the quality of their experiments.

**Figure 8:**
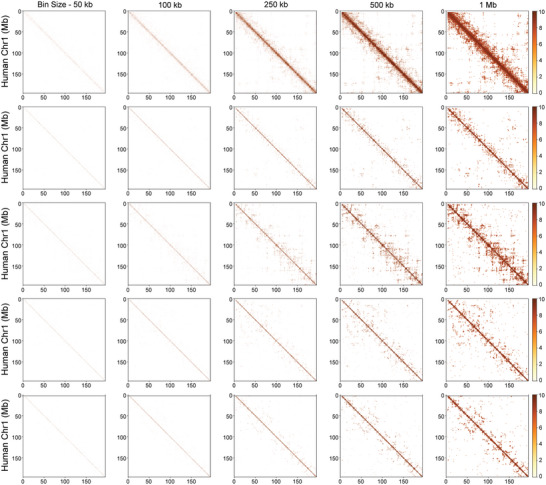
Heatmap of interaction matrices with different bin sizes. The raw counts are shown for chromosome 1 from 5 randomly selected single blastomeres at the 1‐cell stage. All cells display clearer patterns as the bin size increases. The last two cells (fourth and fifth rows) show similar interaction patterns but differ from the other cells.

Although it is widely acknowledged that scHi‐C data are extremely sparse, existing methods do not fully account for this data sparsity by optimizing the scale (i.e., the bin size) used to partition chromosomes for constructing interaction matrices. Currently, all available methods rely on empirically chosen bin sizes (e.g., 100 kb, 500 kb) for constructing contact matrices, but none optimize the best scales for different datasets to achieve precise results.^[^ ^8^, ^10^, ^22^ ^]^ It is worth noting that, interaction matrices generated with different bin sizes (50 kb, 100 kb, 250 kb, 500 kb, and 1 Mb) exhibit variable patterns across cells even within the same cell type (**Figure** **8**). While large bin sizes can reduce noise and computational load, it may overlook finer‐scale chromatin interaction patterns, such as small loops or enhancer‐promoter interactions. Conversely, using very small bin sizes means each bin covers a smaller genomic region, potentially capturing more detailed interactions. However, smaller bins exacerbate data sparsity, meaning that most bins will contain fewer or no interaction counts. Therefore, selecting the optimal bin size is a balance between maintaining sufficient resolution to detect meaningful patterns and avoiding excessive sparsity. Furthermore, the optimal bin size should be tailored to different computational tasks, such as cell clustering, A/B compartment calling, TAD calling, loop calling and 3D reconstruction, as these tasks rely on different features at different signal levels.

### 7.2 Data Imputation

Like other single‐cell technologies, scHi‐C data is plagued by dropouts, making it difficult to distinguish between true structural zeroes (where no interaction exists) and dropouts (where an interaction exists but was not captured). Although many methods exist to impute missing data in scHi‐C, these methods typically rely on information from neighboring genomic locations (Table 4). However, this approach can dilute high‐frequency contacts and result in a high rate of false positives, which may lead to biological artifacts and incorrect interpretations.

To improve the imputation process for scHi‐C data, several strategies can be employed. First, it is valuable to perform cell type‐specific imputation. A robust approach to imputation could involve clustering cells by type before performing data imputation. By imputing data within clusters of similar cells, rather than relying solely on neighboring genomic location signals, this method could better retain structural zeroes while effectively addressing experimental dropouts. These steps could be performed iteratively to further refine the results. Second, it is necessary to utilize advanced deep learning‐based strategies. Several deep learning strategies have already been applied to scHi‐C data imputation. For instance, the variational autoencoder‐based model scVI‐3D integrates scHi‐C data with other omics data for imputation.^[^ ^42^ ^]^ scHiCEDRN uses deep recurrent neural networks for imputing missing scHi‐C data,^[^ ^51^ ^]^ while Higashi utilizes a deep graph embedding model with a transformer‐based architecture.^[^ ^37^ ^]^ Other advanced strategies, such as self‐supervised learning,^[^ ^70^ ^]^ transformer model,^[^ ^71^ ^]^ attention mechanisms,^[^ ^72^ ^]^ which have been successfully used in scRNA‐seq data analysis, could also be utilized for scHi‐C data imputation. By adopting these strategies, the accuracy and reliability of data imputation in scHi‐C analyses could be significantly enhanced.

### 7.3 Data Integration

Combining scHi‐C with other assays, such as RNA‐seq, DNA methylation, ATAC‐seq, or DNA sequencing, has been implemented in various studies.^[^ ^9^, ^10^, ^11^ ^]^ Currently, these data types are often analyzed individually, and researchers link the results from each data type manually. There is a lack of efficient and automated methods for integrative analysis of scHi‐C data with other single‐cell assays by correcting batch effects and standardizing data units. To this end, a promising approach is to design deep learning‐based methods similar to those in the integrative analysis of scRNA‐seq and scATAC‐seq data sets.^[^ ^73^, ^74^, ^75^, ^76^ ^]^ For example, we recently developed DeepBID, a novel deep learning‐based method for batch effect correction, non‐linear dimensionality reduction, embedding and cell clustering concurrently.^[^ ^74^ ^]^ DeepBID utilizes a NB‐based autoencoder with dual Kullback‐Leibler divergence loss functions, aligning cell points from different batches within a consistent low‐dimensional latent space and progressively mitigating batch effects through iterative clustering. This strategy can be further generalized to design multiple autoencoders to hierarchically integrate different batches of one data type and multiple data types. First, we can train batch‐specific autoencoders for each batch to capture batch‐specific variability and mitigate noise. These autoencoders can use NB distributions to handle data sparsity and overdispersion while aligning each batch in a consistent latent space. Second, for multiple data types (e.g., scRNA‐seq and scATAC‐seq), we can use separate autoencoders to learn modality‐specific embeddings. These embeddings can be merged into a shared latent space through a higher‐level autoencoder, using cross‐modal alignment losses to maintain consistency and capture complementary features across modalities.

### 7.4 Comparative Analysis

Given the challenges posed by data sparsity, dropout noise and small sample sizes in scHi‐C datasets, it is crucial to design efficient comparative methods with high statistical power. For instance, scHiCDiff utilizes NB and ZINB distributions to model interaction strengths among chromosomal loci and tests significant interactions by KS or CVM tests.^[^ ^49^ ^]^ However, these non‐parametric testing approaches often suffer from lower statistical power compared to parametric methods, particularly when dealing with small sample sizes. Recent advancements have derived analytical results for the exact distribution of the difference between two NB distributions (DOTNB) and have successfully applied them to scRNA‐seq datasets, demonstrating high performance and enhanced sensitivity in small datasets.^[^ ^77^ ^]^ Incorporating the theoretical advancements of DOTNB into the comparative analysis of scHi‐C data will significantly improve sensitivity, particularly in studies with small sample sizes, thereby enhancing the reliability and reproducibility of results. Another consideration is to design methods for detecting dynamic 3D chromosomal organizations at various scales, such as A/B compartments, TADs, and loops. Instead of the bin‐based comparison strategy in scHiCDiff, statistical models should be carefully developed using their boundary information and interaction strengths to provide accurate testing results.

## 8 Conclusion

The development of scHi‐C and its variants has significantly advanced our understanding of the functional roles of chromatin 3D architectures, yet many challenges remain unresolved. By assessing the quality of available datasets generated by different protocols, we highlighted that hybrid scHi‐C protocols combined with other assays are effective in capturing reasonable numbers of chromatin interactions along with other independent molecular signals. However, scHi‐C data is widely considered to be extremely sparse, which limits its ability to accurately characterize the interaction map of a single cell. Addressing this issue requires a continued focus on the development of novel scHi‐C protocols with higher capture efficiency. Additionally, the application of scHi‐C in biological research remains limited, primarily due to the experimental complexity and high costs. Compared with scRNA‐seq and scATAC, there is a lack of third‐party services for scHi‐C in the current market, and existing services are prohibitively expensive. Therefore, optimized and highly efficient versions of scHi‐C protocols are needed for broader adoption and application.

We also summarized important scHi‐C analysis topics, and the current pipelines designed to answer those computational questions. However, there is not a single pipeline to complete all these computational tasks due to the inherent data sparsity and different experimental protocols. The development of a unified and scalable computational framework that can handle the complexities of scHi‐C data across various experimental conditions is imperative. This framework should be adaptable, allowing for the integration of multiple data types, such as RNA‐seq, ATAC‐seq and DNA methylation data, while also being robust enough to manage the inherent sparsity of scHi‐C datasets. Another important consideration is the standardization of data formats and analysis pipelines, which would allow for better cross‐study comparisons and meta‐analyses. Ultimately, developing community‐driven, open‐source tools that incorporate best practices for quality control, normalization and imputation will be essential for advancing the field. This would enable researchers to derive meaningful insights from scHi‐C data, contributing to a deeper understanding of chromatin organization and its role in gene regulation, cellular dynamics and disease pathogenesis.

All datasets used in this review are publicly available. The GEO accession numbers for the datasets can be found in Table 3. All programming scripts for the data analysis and comparison are available on GitHub https://github.com/chenyongrowan/scHIC_Evaluation.

## Conflict of Interest

The authors declare no conflict of interest.

## Author Contributions

Y.C. conceived the project; M.D. analyzed the data; M.D. and Y.C. wrote the paper.

## References

[1] J. Dekker , Science 2008, 319, 1793. 18369139 10.1126/science.1152850 PMC2666883

[2] J. Dekker , K. Rippe , M. Dekker , N. Kleckner , Science 2002, 295, 1306. 11847345 10.1126/science.1067799

[3] E. Lieberman‐Aiden , N. L. van Berkum , L. Williams , M. Imakaev , T. Ragoczy , A. Telling , I. Amit , B. R. Lajoie , P. J. Sabo , M. O. Dorschner , R. Sandstrom , B. Bernstein , M. A. Bender , M. Groudine , A. Gnirke , J. Stamatoyannopoulos , L. A. Mirny , E. S. Lander , J. Dekker , Science 2009, 326, 289. 19815776 10.1126/science.1181369 PMC2858594

[4] J. R. Dixon , S. Selvaraj , F. Yue , A. Kim , Y. Li , Y. Shen , M. Hu , J. S. Liu , B. Ren , Nature 2012, 485, 376. 22495300 10.1038/nature11082 PMC3356448

[5] S. S. P. Rao , M. H. Huntley , N. C. Durand , E. K. Stamenova , I. D. Bochkov , J. T. Robinson , A. L. Sanborn , I. Machol , A. D. Omer , E. S. Lander , E. L. Aiden , Cell 2014, 159, 1665. 25497547 10.1016/j.cell.2014.11.021 PMC5635824

[6] T. Nagano , Y. Lubling , T. J. Stevens , S. Schoenfelder , E. Yaffe , W. Dean , E. D. Laue , A. Tanay , P. Fraser , Nature 2013, 502, 59. 24067610 10.1038/nature12593 PMC3869051

[7] L. Tan , D. Xing , C.‐H. Chang , H. Li , X. S. Xie , Science 2018, 361, 924. 30166492 10.1126/science.aat5641 PMC6360088

[8] V. Ramani , X. Deng , R. Qiu , K. L. Gunderson , F. J. Steemers , C. M. Disteche , W. S. Noble , Z. Duan , J. Shendure , Nat. Methods 2017, 14, 263. 28135255 10.1038/nmeth.4155 PMC5330809

[9] X. Wen , Z. Luo , W. Zhao , R. Calandrelli , T. C. Nguyen , X. Wan , J. L. Charles Richard , S. Zhong , Nature 2024, 628, 648. 38538789 10.1038/s41586-024-07239-w PMC11023937

[10] D.‐S. Lee , C. Luo , J. Zhou , S. Chandran , A. Rivkin , A. Bartlett , J. R. Nery , C. Fitzpatrick , C. O'Connor , J. R. Dixon , J. R. Ecker , Nat. Methods 2019, 16, 999. 31501549 10.1038/s41592-019-0547-z PMC6765423

[11] G. Li , Y. Liu , Y. Zhang , N. Kubo , M. Yu , R. Fang , M. Kellis , B. Ren , Nat. Methods 2019, 16, 991. 31384045 10.1038/s41592-019-0502-z PMC6765429

[12] Z. Liu , Y. Chen , Q. Xia , M. Liu , H. Xu , Y. Chi , Y. Deng , D. Xing , Science 2023, 380, 1070. 37289875 10.1126/science.adg3797

[13] L. Chang , Y. Xie , B. Taylor , Z. Wang , J. Sun , E. J. Armand , S. Mishra , J. Xu , M. Tastemel , A. Lie , Z. A. Gibbs , H. S. Indralingam , T. M. Tan , R. Bejar , C. C. Chen , F. B. Furnari , M. Hu , B. Ren , Nat. Biotechnol. 2024. 10.1038/s41587-024-02447-1 PMC12520981 39424717

[14] Y. Wang , N. E. Navin , Mol. Cell 2015, 58, 598. 26000845 10.1016/j.molcel.2015.05.005 PMC4441954

[15] T. Nagano , Y. Lubling , C. Várnai , C. Dudley , W. Leung , Y. Baran , N. Mendelson Cohen , S. Wingett , P. Fraser , A. Tanay , Nature 2017, 547, 61. 28682332 10.1038/nature23001 PMC5567812

[16] A. Wei , H. Zhang , Q. Qiu , E. B. Fabyanic , P. Hu , H. Wu , bioRxiv 2023, 529096.

[17] C. Luo , H. Liu , F. Xie , E. J. Armand , K. Siletti , T. E. Bakken , R. Fang , W. I. Doyle , T. Stuart , R. D. Hodge , L. Hu , B.‐A. Wang , Z. Zhang , S. Preissl , D.‐S. Lee , J. Zhou , S.‐Y. Niu , R. Castanon , A. Bartlett , A. Rivkin , X. Wang , J. Lucero , J. R. Nery , D. A. Davis , D. C. Mash , R. Satija , J. R. Dixon , S. Linnarsson , E. Lein , M. M. Behrens , et al., Cell Genom. 2022, 2, 100107. 35419551 10.1016/j.xgen.2022.100107 PMC9004682

[18] H. Wu , J. Zhang , F. Jian , J. P. Chen , Y. Zheng , L. Tan , X. Sunney Xie , Nat. Methods 2024, 21, 974. 38622459 10.1038/s41592-024-02239-0 PMC11166570

[19] H. Liu , Q. Zeng , J. Zhou , A. Bartlett , B.‐A. Wang , P. Berube , W. Tian , M. Kenworthy , J. Altshul , J. R. Nery , H. Chen , R. G. Castanon , S. Zu , Y. E. Li , J. Lucero , J. K. Osteen , A. Pinto‐Duarte , J. Lee , J. Rink , S. Cho , N. Emerson , M. Nunn , C. O'Connor , Z. Wu , I. Stoica , Z. Yao , K. A. Smith , B. Tasic , C. Luo , J. R. Dixon , et al., Nature 2023, 624, 366. 38092913 10.1038/s41586-023-06805-y PMC10719113

[20] I. M. Flyamer , J. Gassler , M. Imakaev , H. B. Brandão , S. V. Ulianov , N. Abdennur , S. V. Razin , L. A. Mirny , K. Tachibana‐Konwalski , Nature 2017, 544, 110. 28355183 10.1038/nature21711 PMC5639698

[21] N. Rappoport , E. Chomsky , T. Nagano , C. Seibert , Y. Lubling , Y. Baran , A. Lifshitz , W. Leung , Z. Mukamel , R. Shamir , P. Fraser , A. Tanay , Nat. Commun. 2023, 14, 3844. 37386027 10.1038/s41467-023-39549-4 PMC10310791

[22] T. J. Stevens , D. Lando , S. Basu , L. P. Atkinson , Y. Cao , S. F. Lee , M. Leeb , K. J. Wohlfahrt , W. Boucher , A. O'Shaughnessy‐Kirwan , J. Cramard , A. J. Faure , M. Ralser , E. Blanco , L. Morey , M. Sansó , M. G. S. Palayret , B. Lehner , L. Di Croce , A. Wutz , B. Hendrich , D. Klenerman , E. D. Laue , Nature 2017, 544, 59. 28289288 10.1038/nature21429 PMC5385134

[23] W. Li , J. Lu , P. Lu , Y. Gao , Y. Bai , K. Chen , X. Su , M. Li , J.'e Liu , Y. Chen , L. Wen , F. Tang , Nat. Methods 2023, 20, 1493. 37640936 10.1038/s41592-023-01978-w

[24] S. Zhou , W. Jiang , Y. Zhao , D.‐X. Zhou , Nat Plants 2019, 5, 795. 31332313 10.1038/s41477-019-0471-3

[25] V. Ramani , X. Deng , R. Qiu , C. Lee , C. M. Disteche , W. S. Noble , J. Shendure , Z. Duan , Methods 2020, 170, 61. 31536770 10.1016/j.ymeth.2019.09.012 PMC6949367

[26] M. V. Arrastia , J. W. Jachowicz , N. Ollikainen , M. S. Curtis , C. Lai , S. A. Quinodoz , D. A. Selck , R. F. Ismagilov , M. Guttman , Nat. Biotechnol. 2022, 40, 64. 34426703 10.1038/s41587-021-00998-1 PMC11588347

[27] R. M. Mulqueen , D. Pokholok , B. L. O'Connell , C. A. Thornton , F. Zhang , B. J. O'Roak , J. Link , G. G. Yardimci , R. C. Sears , F. J. Steemers , A. C. Adey , Nat. Biotechnol. 2021, 39, 1574. 34226710 10.1038/s41587-021-00962-z PMC8678206

[28] T. Zhou , R. Zhang , J. Ma , Annu. Rev. Biomed. Data Sci. 2021, 4, 21. 34465168 10.1146/annurev-biodatasci-020121-084709

[29] M. G. Dozmorov , K. M. Tyc , N. C. Sheffield , D. C. Boyd , A. L. Olex , J. Reed , J. C. Harrell , Gigascience 2021, 10, giab022. 33880552 10.1093/gigascience/giab022 PMC8058593

[30] P. Hansen , Genes (Basel) 2019, 10, 43.30646598

[31] H.‐J. Kim , G. G. Yardimci , G. Bonora , V. Ramani , J. Liu , R. Qiu , C. Lee , J. Hesson , C. B. Ware , J. Shendure , Z. Duan , W. S. Noble , PLoS Comput. Biol. 2020, 16, e1008173. 32946435 10.1371/journal.pcbi.1008173 PMC7526900

[32] J. Gassler , H. B. Brandão , M. Imakaev , I. M. Flyamer , S. Ladstätter , W. A. Bickmore , J.‐M. Peters , L. A. Mirny , K. Tachibana , EMBO J. 2017, 36, 3600. 29217590 10.15252/embj.201798083 PMC5730859

[33] S. V. Ulianov , V. V. Zakharova , A. A. Galitsyna , P. I. Kos , K. E. Polovnikov , I. M. Flyamer , E. A. Mikhaleva , E. E. Khrameeva , D. Germini , M. D. Logacheva , A. A. Gavrilov , A. S. Gorsky , S. K. Nechaev , M. S. Gelfand , Y. S. Vassetzky , A. V. Chertovich , Y. Y. Shevelyov , S. V. Razin , Nat. Commun. 2021, 12, 41. 33397980 10.1038/s41467-020-20292-z PMC7782554

[34] L. Tan , D. Xing , N. Daley , X. S. Xie , Nat. Struct. Mol. Biol. 2019, 26, 297. 30936528 10.1038/s41594-019-0205-2

[35] L. Tan , W. Ma , H. Wu , Y. Zheng , D. Xing , R. Chen , X. Li , N. Daley , K. Deisseroth , X. S. Xie , Cell 2021, 184, 741. 33484631 10.1016/j.cell.2020.12.032

[36] T. Liu , Z. Wang , Genes (Basel) 2022, 13. 10.3390/genes13061048 PMC9222580 35741810

[37] R. Zhang , T. Zhou , J. Ma , Nat. Biotechnol. 2022, 40, 254. 34635838 10.1038/s41587-021-01034-y PMC8843812

[38] S. Fan , D. Dang , Y. Ye , S.‐W. Zhang , L. Gao , S. Zhang , J. Mol. Cell Biol. 2023, 15, mjad003. 36708167 10.1093/jmcb/mjad003 PMC10308180

[39] C. A. Horton , B. H. Alver , P. J. Park , Bioinformatics 2020, 36, 2902. 32003786 10.1093/bioinformatics/btaa048 PMC8453230

[40] J. Wolff , L. Rabbani , R. Gilsbach , G. Richard , T. Manke , R. Backofen , B. A. Grüning , Nucleic Acids Res. 2020, 48, W177. 32301980 10.1093/nar/gkaa220 PMC7319437

[41] T. Liu , Z. Wang , Bioinformatics 2018, 34, 1046. 29186290 10.1093/bioinformatics/btx747 PMC5860379

[42] Y. Zheng , S. Shen , S. Keleş , Genome Biol. 2022, 23, 222. 36253828 10.1186/s13059-022-02774-z PMC9575231

[43] J. Zhou , J. Ma , Y. Chen , C. Cheng , B. Bao , J. Peng , T. J. Sejnowski , J. R. Dixon , J. R. Ecker , Proc. Natl. Acad. Sci. USA 2019, 116, 14011. 31235599 10.1073/pnas.1901423116 PMC6628819

[44] R. Zhang , T. Zhou , J. Ma , Cell Syst. 2022, 13, 798. 36265466 10.1016/j.cels.2022.09.004 PMC9867958

[45] X. Li , L. Lee , A. Abnousi , M. Yu , W. Liu , L. Huang , Y. Li , M. Hu , Comput. Struct. Biotechnol. J. 2022, 20, 2778. 35685374 10.1016/j.csbj.2022.05.046 PMC9168059

[46] Y. Ye , S. Zhang , L. Gao , Y. Zhu , J. Zhang , Adv. Sci. (Weinh) 2023, 10, e2205162. 36658736 10.1002/advs.202205162 PMC10015865

[47] X. Li , F. Feng , H. Pu , W. Y. Leung , J. Liu , PLoS Comput. Biol. 2021, 17, e1008978. 34003823 10.1371/journal.pcbi.1008978 PMC8162587

[48] Y. Zhou , C. Wu , L. Tan , Phys. A: Statis. Mechanics Appl. 2021, 570, 125783.

[49] H. Liu , W. Ma , Bioinformatics 2023, 39. 10.1093/bioinformatics/btad625 PMC10598576 37847655

[50] Q. Xie , C. Han , V. Jin , S. Lin , PLoS Comput. Biol. 2022, 18, e1010129. 35696429 10.1371/journal.pcbi.1010129 PMC9232133

[51] Y. Wang , Z. Guo , J. Cheng , Bioinformatics 2023, 39, btad458. 37498561 10.1093/bioinformatics/btad458 PMC10403428

[52] C. Zhen , Brief. Bioinformat. 2022, 23.

[53] J. R. Dixon , I. Jung , S. Selvaraj , Y. Shen , J. E. Antosiewicz‐Bourget , A. Y. Lee , Z. Ye , A. Kim , N. Rajagopal , W. Xie , Y. Diao , J. Liang , H. Zhao , V. V. Lobanenkov , J. R. Ecker , J. A. Thomson , B. Ren , Nature 2015, 518, 331. 25693564 10.1038/nature14222 PMC4515363

[54] E. M. Hildebrand , J. Dekker , Trends Biochem. Sci. 2020, 45, 385. 32311333 10.1016/j.tibs.2020.01.002 PMC7275117

[55] Y. Chen , Y. Wang , Z. Xuan , M. Chen , M. Q. Zhang , Nucleic Acids Res. 2016, 44, e106. 27060148 10.1093/nar/gkw225 PMC4914103

[56] I. Boltsis , F. Grosveld , G. Giraud , P. Kolovos , Front Cell Dev Biol 2021, 9, 723859. 34422840 10.3389/fcell.2021.723859 PMC8371409

[57] K. Xiong , R. Zhang , J. Ma , Nat. Methods 2024, 21, 814. 38589516 10.1038/s41592-024-02230-9 PMC11127718

[58] Q. Szabo , F. Bantignies , G. Cavalli , Sci. Adv. 2019, 5, eaaw1668. 30989119 10.1126/sciadv.aaw1668 PMC6457944

[59] J. Dekker , E. Heard , FEBS Lett. 2015, 589, 2877. 26348399 10.1016/j.febslet.2015.08.044 PMC4598308

[60] E. McArthur , J. A. Capra , Am. J. Hum. Genet. 2021, 108, 269. 33545030 10.1016/j.ajhg.2021.01.001 PMC7895846

[61] M. Zufferey , D. Tavernari , E. Oricchio , G. Ciriello , Genome Biol. 2018, 19, 217. 30526631 10.1186/s13059-018-1596-9 PMC6288901

[62] A. Li , G. Zeng , H. Wang , X. Li , Z. Zhang , Adv. Sci. (Weinh) 2023, 10, e2300366. 37162225 10.1002/advs.202300366 PMC10369259

[63] J. T. Sanders , R. Golloshi , P. Das , Y. Xu , P. H. Terry , D. G. Nash , J. Dekker , R. P. McCord , Sci. Rep. 2022, 12, 4721. 35304523 10.1038/s41598-022-08602-5 PMC8933507

[64] A. S. Hansen , I. Pustova , C. Cattoglio , R. Tjian , X. Darzacq , Elife 2017, 6, e25776. 28467304 10.7554/eLife.25776 PMC5446243

[65] S. Kadauke , G. A. Blobel , Biochim. Biophys. Acta 2009, 1789, 17. 18675948 10.1016/j.bbagrm.2008.07.002 PMC2638769

[66] L. Meng , C. Wang , Y. Shi , Q. Luo , Nat. Commun. 2021, 12, 4369. 34272403 10.1038/s41467-021-24662-z PMC8285481

[67] P. I. Kos , A. A. Galitsyna , S. V. Ulianov , M. S. Gelfand , S. V. Razin , A. V. Chertovich , PLoS Comput. Biol. 2021, 17, e1009546. 34793453 10.1371/journal.pcbi.1009546 PMC8601426

[68] H. Lyu , E. Liu , Z. Wu , Y. Li , Y. Liu , X. Yin , Bioinformatics 2022, 38, 5151. 36205615 10.1093/bioinformatics/btac670

[69] R. G. Gallager , Stochastic Processes: Theory for Applications, Cambridge University Press, Cambridge 2013.

[70] T. Lei , R. Chen , S. Zhang , Y. Chen , Brief. Bioinform. 2023, 24, bbad335. 37769630 10.1093/bib/bbad335 PMC10539043

[71] C. Zhao , Z. Xu , X. Wang , S. Tao , W. A. MacDonald , K. He , A. C. Poholek , K. Chen , H. Huang , W. Chen , Brief. Bioinform. 2024, 25, bbae052. 38436557 10.1093/bib/bbae052 PMC10939304

[72] Y. Cheng , X. Ma , Bioinformatics 2022, 38, 2187. 35176138 10.1093/bioinformatics/btac099

[73] T. Athaya , R. C. Ripan , X. Li , H. Hu , Brief. Bioinform. 2023, 24. 10.1093/bib/bbad313 PMC10516349 37651607

[74] L. Qin , G. Zhang , S. Zhang , Y. Chen , Adv. Sci. (Weinh) 2024, e2308934. 38778573 10.1002/advs.202308934 PMC11304254

[75] M. D. Luecken , M. Büttner , K. Chaichoompu , A. Danese , M. Interlandi , M. F. Mueller , D. C. Strobl , L. Zappia , M. Dugas , M. Colomé‐Tatché , F. J. Theis , Nat. Methods 2022, 19, 41. 34949812 10.1038/s41592-021-01336-8 PMC8748196

[76] Y. Zhou , Q. Sheng , J. Qi , J. Hua , B. Yang , L. Wan , S. Jin , Genome Res. 2023, 33, 750. 37308294 10.1101/gr.277522.122 PMC10317120

[77] A. Petrany , R. Chen , S. Zhang , Y. Chen , Genome Res. 2024, 34, 1636. 39406498 10.1101/gr.278843.123 PMC11529838

